# Antimicrobial resistance and zinc resistance gene *czrC* prevalence differs among methicillin resistant *Staphylococcus aureus* clonal complex 5 isolates from health care and agricultural sources

**DOI:** 10.3389/fmicb.2026.1977429

**Published:** 2026-09-24

**Authors:** Tracy L. Nicholson, Sarah M. Shore

**Affiliations:** National Animal Disease Center, Agricultural Research Service, USDA, Ames, IA, United States

**Keywords:** *Staphylococcus aureus*, MRSA, AMR, CC5, swine, ST5, LA-MRSA

## Abstract

Since the discovery of livestock-associated methicillin-resistant *Staphylococcus aureus* (MRSA), public health concerns have grown surrounding the potential of livestock-associated MRSA (LA-MRSA) isolates to serve as a reservoir to disseminate antimicrobial resistance beyond agricultural settings. To further address concerns, this study evaluated human clinical MRSA CC5 isolates obtained from swine-dense regions for *czrC* gene prevalence as well as antimicrobial resistance prevalence and diversity. These findings were compared with those from human clinical MRSA CC5 isolates obtained from individuals with no swine contact and swine-associated LA-MRSA CC5 isolates. Across all three isolate subgroups, the *czrC* gene was detected at a low frequency, further supporting the notion that zinc resistance in MRSA is largely influenced by lineage-specific factors. Swine-associated LA-MRSA CC5 isolates exhibited resistance to fewer antimicrobial agents than the human-associated isolate subgroups. Moreover, human-associated and swine-associated CC5 isolates harbored distinct antimicrobial resistance genetic determinants, including differences in plasmid-borne resistance elements. Collectively, these findings demonstrate that, among the CC5 isolates examined, human and swine-associated CC5 MRSA represent distinct antimicrobial resistance reservoirs, suggesting that the swine reservoir is unlikely to serve as a significant source of antimicrobial resistance dissemination beyond the agricultural sector.

## Introduction

Methicillin-resistant *Staphylococcus aureus* (MRSA) is a substantial contributor to the global antimicrobial resistance (AMR) burden, and is associated with increased morbidity, mortality, and healthcare costs (Algammal et al., 2020; Maragakis et al., 2008). The vast majority of AMR genes harbored by MRSA isolates are carried on mobile genetic elements (MGEs). The most notable of the MGEs is the SCC*mec* element harboring *mecA*, or less commonly *mecB* or *mecC*, which encodes resistance to methicillin and other β-lactam antimicrobials (Nunes Nascimento et al., 2026; Jevons, 1961; Deurenberg and Stobberingh, 2008).

Multi-locus sequence typing (MLST) and staphylococcal cassette chromosome *mec* (SCC*mec*) typing are molecular techniques commonly used to track the spread of specific isolates, determine the clonal relationship among isolates, and establish if an outbreak originated in a hospital setting, a community setting, or from animal contact. MLST uses allele profiles based on the sequence of seven housekeeping genes to determine sequence type (ST). Isolates with different STs are further grouped together into a clonal complex (CC) when the isolates share at least five of the seven alleles (Enright et al., 2000). SCCmec typing differentiates isolates based on the sequencing of the *mec* gene complexes and *ccr* gene complexes, which is beneficial when discriminating between isolates with identical STs (Milheirico et al., 2007; Ito et al., 2001).

MRSA isolates are epidemiologically grouped based on their source into hospital-acquired (HA), community-acquired (CA), or livestock associated (LA) (Deurenberg and Stobberingh, 2008; Naimi et al., 2003; Stefani et al., 2012). In general, infections occurring from HA-MRSA isolates are associated with hospital settings and a predisposing factor or illness (Deurenberg and Stobberingh, 2008; Naimi et al., 2003; DeLeo et al., 2010). Conversely, infections due to CA-MRSA isolates typically occur in healthy humans outside of health care settings and environments. For this reason, CA-MRSA isolates are considered more virulent than HA-MRSA isolates, mainly due to the variety of toxins they harbor (Deurenberg and Stobberingh, 2008; Naimi et al., 2003; DeLeo et al., 2010; David and Daum, 2010). LA-MRSA was first reported in swine herds in 2005 and raised public health concerns that swine and other livestock species could potentially serve as a MRSA reservoir and source for human infections (Voss et al., 2005). Subsequent research has shown that LA-MRSA isolates do not carry genes encoding human specific virulence factors and are therefore regarded as less capable of causing human infections (Hau et al., 2015; Price et al., 2012; Pantosti, 2012). However, LA-MRSA isolates remain a top public health research priority since LA-MRSA isolates are the largest reservoir of MRSA outside of hospital settings and have been shown to harbor numerous AMR genes (Pantosti, 2012; Challagundla et al., 2018; Schijffelen et al., 2010; Monecke et al., 2011).

A high prevalence of zinc resistance among swine-associated LA-MRSA isolates from Europe and North America has been reported (Price et al., 2012; Cavaco et al., 2010; Slifierz et al., 2015; Cavaco et al., 2011). Dietary zinc supplementation is commonly used in weaned pigs to control enteric disease (Fairbrother et al., 2005). Because the *czrC* gene, encoding zinc and cadmium resistance, and *mecA* are both co-located on the SCC*mec* element, it has been suggested that the use of in feed zinc as an antidiarrheal agent has the potential to contribute to the emergence and spread of LA-MRSA isolates in livestock environments through increased selection pressure to maintain the SCC*mec* element (Fairbrother et al., 2005; Aarestrup et al., 2010; Slifierz et al., 2014). While recent data has demonstrated that *czrC* gene prevalence is associated with ST lineage, indicating that co-selection associated with in-feed zinc supplementation has not contributed to the persistence or prevalence of LA-MRSA isolates within swine herds, continued evaluation and monitoring of *czrC* gene prevalence among MRSA isolates remains a high priority for research interest (Hau et al., 2017).

Studies have demonstrated that LA-MRSA lineage prevalence varies based on region. For example, ST398 is the most common lineage in European swine populations, while ST9 is the most common lineage in Asian countries (Wagenaar et al., 2009). A more diverse lineage prevalence including ST398, ST9, and ST5 lineages has been reported for swine herds in the United States (Molla et al., 2012; Frana et al., 2013; Sun et al., 2015). Unlike ST9 and ST398 MRSA isolates, which are considered livestock adapted and typically do not cause infections in humans, ST5 isolates are a globally disseminated and highly successful lineage that includes both CA- and HA-MRSA clones reaching pandemic levels (Pantosti, 2012; Challagundla et al., 2018; Monecke et al., 2011; Becker et al., 2015). The global success and predominance of the ST5 and CC5 lineage has been linked to the ability of the lineage to acquire MGEs carrying genes encoding virulence factors or AMR (Pantosti, 2012; Challagundla et al., 2018; Monecke et al., 2011; Fitzgerald, 2012). The prevalence of ST5 isolates in United States swine herds has led to increased public health concerns over the potential for LA-MRSA isolates to disseminate AMR beyond agricultural settings.

A previous study investigated the prevalence and diversity of AMR phenotypes and genetic determinants conferring AMR between LA-MRSA ST5 isolates from a variety of swine associated sources and clinical MRSA ST5 isolates from humans with no swine contact. The results found separate and distinct genetic AMR elements were harbored by each subgroup, with little to no overlap in shared AMR genes between the swine associated LA-MRSA ST5 and clinical MRSA ST5 isolates (Hau et al., 2018a). To fully evaluate the potential of LA-MRSA CC5 isolates to serve as a potential source for MGEs harboring AMR, follow up studies that include human MRSA isolates from regions of dense swine production are needed. Toward that effort, in this report human clinical MRSA CC5 isolates obtained from swine-dense regions were evaluated for *czrC* gene prevalence and the prevalence and diversity of AMR phenotypes and AMR genetic elements. These results were then compared to the *czrC* gene prevalence, phenotypic resistances, and AMR genetic elements harbored among human clinical MRSA CC5 isolates obtained from humans with no swine contact and swine-associated LA-MRSA CC5 isolates.

## Materials and methods

### Isolates and culture conditions

Human clinical isolates were obtained from the Iowa State Hygienic Laboratory submitted from routine diagnostic cases occurring throughout the state of Iowa. Iowa is the most swine-dense and top pork-producing state in the United States, containing approximately 24.5 million hogs and approximately one-third of the entire United States pig population (Hayes, 2026). After CC determination, only CC5 clinical MRSA isolates obtained from swine dense regions (clinical MRSA CC5-SDR; *n* = 108) were included in the study and further analyzed (Supplementary Table 1). The STs of the clinical MRSA CC5-SDR isolates included in the study were ST5 (*n* = 80), ST105 (*n* = 19), ST225 (*n* = 2), ST231 (*n* = 4), ST6170 (*n* = 2), and ST6171 (*n* = 1) (Supplementary Table 1). Frozen stocks (−80 °C in 30% glycerol) of isolates were streaked onto Trypticase Soy Agar (TSA) (BD Biosciences, Sparks, MD) at 37 °C to obtain isolated colonies. Individual colonies were selected to start an overnight culture of Trypticase Soy Broth (TSB) (BD Biosciences, Sparks, MD). Previously characterized clinical MRSA CC5 isolates obtained from humans with no swine contact (clinical MRSA CC5-NSC) and swine associated LA-MRSA CC5 isolates (LA-MRSA CC5) were included in the analyses (Supplementary Table 1) (Hau et al., 2018a). All isolates were either obtained from samples collected as part of previous studies or were obtained from samples submitted as part of field case investigations and did not require Institutional Animal Care and Use Committee (IACUC) approval or Institutional Review Board (IRB) approval.

### Whole-genome sequencing, assembly, and annotation

Strains were grown overnight on TSA (BD Biosciences, Sparks, MD) at 37 °C to obtain isolated colonies. Individual colonies were selected to and cultured overnight in TSB (BD Biosciences, Sparks, MD). After 12–18 h of growth, 750 μL of the liquid culture was pelleted and the supernatant was removed. The pelleted cells were stored at −80 °C until DNA extraction. To isolate DNA, each pellet was resuspended in 200 μL 1 × Phosphate Buffered Saline with 0.2 M EDTA. To lyse the cells, the following were added to each suspension: 12 μL of Lysozyme solution (Sigma, St. Louis, MO), 1 μL RNase (Roche, Mannheim, Germany), 7.5 μL Lysostaphin solution (Sigma, St. Louis, MO), and 7.5 μL Mutanolysin solution (Sigma, St. Louis, MO). The cells were then incubated for 1 h at 37 °C. Forty microliters of Proteinase K (Roche, Mannheim, Germany) was added and the suspension was incubated overnight at 55 °C. The following day, a Roche High Pure PCR Template Preparation Kit (Roche, Mannheim, Germany) was used to isolate DNA according to the manufacturer’s protocol. The Elution Buffer containing DNA samples was centrifuged for 5 min at 8000×g to remove visible debris. The Qubit dsDNA BR Assay Kit (Life Technologies, Eugene, OR) was used to determine DNA concentration. WGS assemblies for isolates were obtained using Illumina short read data.

Illumina HiSeq data was obtained from a library created using the NEBNext Ultra II DNA Library Prep Kit (New England Biolabs, Ipswich, MA) sequenced on a HiSeq 3000 instrument generating 2 × 100 bp paired-end reads. Sequencing reads were assessed for quality using FastQC^1^. Reads were randomly subsampled using seqtk^2^ to target a genome coverage of 150X based on an average expected genome length of 2.8 Mbases. Sequence data was assembled using MIRA v. 4.9.6^3^ (Chevreux et al., 1999). The average coverage obtained for each isolate is listed in Supplementary Table 1. To be retained in an assembly, contigs were required to be >1,500 bp in length and have a coverage of >66% of the average coverage for the genome. The assembly tool identified repetitive elements that were required to have a contig length >2000 bp to remain in the assembly. Unless specified otherwise, default parameters were used for all software. Final annotations were completed using NCBI’s Prokaryotic Genome Annotation Pipeline (PGAP) v 4.12 (Tatusova et al., 2016). Accession numbers and genome statistics are summarized in Supplementary Table 1.

### Comparative genomic analysis

Multi-locus sequence typing (MLST) was performed *in silico* on the assembled genome sequences using the MLST software and the PubMLST database^4^ (Jolley et al., 2018; Seemann, 2014). Allele sequences that were missing from an assembly or did not match an existing database sequence were confirmed by PCR using primers developed by Enright et al. and Sanger sequencing (Enright et al., 2000). CC assignments were made utilizing criteria from PubMLST.org for *S. aureus*. SCCmec typing was completed *in silico* on the assembled genome sequences using the primer scheme developed by Zhang et al. (2005). Presence of the β-hemolysin converting bacteriophage was determined *in silico* using Geneious Prime 2025.0.2 (Biomatters Ltd., Auckland, New Zealand) and primers described in Hau et al. (2015). Presence of the *czrC* gene was determined *in silico* using Geneious Prime 2025.0.2 (Biomatters Ltd., Auckland, New Zealand) and primers described in Hau et al. (2017).

### Genomic AMR analysis

An *in silico* search for AMR genes was performed using AMRFinderPlus v.3.12.8 with database version 2024-05-02.2 (Feldgarden et al., 2019). The input option—nucleotide was used to analyze the assembled genome FASTA sequences with default settings. AMR genetic elements were analyzed using Geneious Prime 2025.0.2 (Biomatters Ltd., Auckland, New Zealand). Multiple sequence alignments were used to compare sequence identity of genes and plasmids from different isolates and Geneious Prime 2025.0.2 (Biomatters Ltd., Auckland, New Zealand) was used for image generation. AMR genetic elements harbored by each isolate were utilized to generate a presence/absence binary table, which was converted into a distance matrix heatmap clustered by hierarchical clustering using a complete linkage method with Euclidean distance using the ComplexHeatmap package in R (Gu et al., 2016).

### Phenotypic AMR analysis

Phenotypic antibiotic resistance was determined using the broth microdilution method by National Veterinary Services Laboratories (Ames, IA) following standard operating procedures. Minimum inhibitory concentrations (MICs) were determined for each isolate using the Trek BOPO6F plate (Thermo Fisher Scientific Inc., Oakwood Village, OH) and the Trek GPALL1F plate (Thermo Fisher Scientific Inc., Oakwood Village, OH) with *S. aureus* 29,213 (ATCC, Manassas, VA) serving as the control strain. MICs were evaluated in accordance with Clinical Laboratory Standards Institute (CLSI) recommendations based on Vet01-A4 and M100 25th edition standards to give resistance interpretations (Clinical and Laboratory Standards Institute, 2024a,b) (Table 1) (Supplementary Table 1). AMR index, defined as the proportion of antibiotics tested to which an isolate exhibited phenotypic resistance relative to the number of antibiotics tested, was determined for each isolate using the results of the microbroth dilution analysis.

**Table 1:** *czrC* gene prevalence among isolate groups.

Gene prevalence	Clinical MRSA CC5-SDR	Clinical MRSA CC5-NSC	LA-MRSA CC5
*czrC* PCR prevalence	1/108 (0.9%)	16/71 (22.5%)^a^	0/82 (0%)

a Denotes statistical significance at *p* < 0.0001 as compared to LA-MRSA CC5.

### Statistical analysis

Statistical analysis was completed using GraphPad Prism 10.4.1 (GraphPad Software, Inc., La Jolla, CA). Differences in phenotypic antimicrobial resistance between groups were assessed using Fisher’s exact test. Differences in AMR index and resistance gene numbers were also were assessed using Fisher’s exact test. Results were considered significant using a *p*-value cutoff of *p* < 0.05.

### Data availability

The genome assemblies and sequencing read data have been deposited at DDBJ/ENA/GenBank under BioProject accession number PRJNA645203 and PRJNA645197. Detailed information regarding assembly statistics, BioSample, GenBank, and SRA accession numbers is provided in Supplementary Table 1.

## Results

### *czrC* gene prevalence

Human clinical MRSA CC5-SDR isolates were screened using *czrC*-specific *in-silico* PCR and only one isolate harbored the *czrC* gene (Table 1). The low *czrC* gene prevalence (1/108; 0.9%) among clinical MRSA CC5-SDR was similar to *czrC* gene prevalence among LA-MRSA CC5 isolates (0/82; 0%). In contrast, over one-fifth of the clinical MRSA CC5-NSC isolates (16/71, 22.5%) contained the *czrC* gene, which was significantly higher than the *czrC* gene prevalence among LA-MRSA CC5 isolates (*p* < 0.0001) (Table 1). When *czrC* gene prevalence among the isolate groups was further separated into SCC*mec* element types, the *czrC* gene was only observed among the CC5 isolates that contained the SCC*mec* type II (Table 2). Further examination of the *SCCmec* type II element in the one clinical MRSA CC5-SDR isolate that harbored the *czrC* gene indicated that the *czrC* gene was located upstream of the SCCmec element and possibly transferred with the *speG* gene. The position of the czrC gene in this strain being located upstream of the *SCCmec* element is similar to the location of the *czrC* gene for all clinical MRSA CC5-NSC isolates that harbored the *czrC* gene.

**Table 2:** *czrC* gene prevalence among SCC*mec* types.

Isolate group	SCC*mec* type	*czrC* prevalence
Clinical MRSA CC5-SDR	II	1/106
	IV^a^	0/1
	NT^a^	0/1
Clinical MRSA CC5-NSC	II	69/71
	IV	0/2
LA-MRSA CC5	III	0/17
	IV	0/42
	NT^a^	0/23

a Denotes isolates that were not typable (NT) because they could not be classified into a SCC*mec* type due to the mec complex unable to be determined using available primer sets.

### Phenotypic AMR distribution

Phenotypic resistance was determined for the human clinical MRSA CC5-SDR isolates and subsequently compared to the phenotypic resistances among human clinical MRSA CC5-NSC and LA-MRSA CC5 isolates (Table 3). In general, the phenotypic resistance observed for the clinical MRSA CC5-SDR isolates toward all antibiotics tested was similar to the phenotypic resistance observed for the human clinical MRSA CC5-NSC isolates with three exceptions. These three exceptions include florfenicol, sulfadimethoxine, and clindamycin phenotypic resistance where phenotypic AMR variance was statistically different among all three isolate subgroups (Table 3).

**Table 3:** Phenotypic AMR prevalence among isolate groups.

Antibiotic class	Antibiotic	Clinical MRSA CC5-SDR^a^	Clinical MRSA CC5-NSC^a^	LA-MRSA CC5^a^	Statistics^b^
β-lactam	Penicillin	108/108 (100%)	71/71 (100%)	82/82 (100%)	NS
	Ampicillin	108/108 (100%)	71/71 (100%)	82/82 (100%)	NS
	Oxacillin	108/108 (100%)^c^	71/71 (100%)^d^	78/82 (95.1%)^c,d^	*p* = 0.0333^c,d^
	Cefoxitin	105/108 (97.2%)	71/71 (100%)^c^	74/82 (90.2%)^c^	*p* = 0.0075^c^
	Ceftiofur	105/108 (97.2%)^c^	67/71 (94.4%)	73/82 (89%)^c^	*p* = 0.0324^c^
Aminoglycoside	Gentamicin	2/108 (1.9%)^c,d^	12/71 (16.9%)^d^	22/82 (26.8%)^c^	*p* < 0.001^c^; *p* = 0.003^d^
	Neomycin	90/108 (83.3%)	67/71 (94.4%)	69/82 (84.1%)	NS
	Streptomycin	0/108 (0%)	1/71 (1.4%)	1/82 (1.2%)	NS
Tetracycline	Chlortetracycline	2/108 (1.9%)^c^	0/71 (0%)^d^	65/82 (79.3%)^c,d^	*p* < 0.0001^c,d^
	Oxytetracycline	2/108 (1.9%)^c^	0/71 (0%)^d^	65/82 (79.3%)^c,d^	*p* < 0.0001^c,d^
	Tetracycline	2/108 (1.9%)^c^	0/71 (0%)^d^	65/82 (79.3%)^c,d^	*p* < 0.0001^c,d^
Phenicols	Chloramphenicol	3/108 (2.8%)^c^	1/71 (1.4%)^d^	9/82 (11%)^c,d^	*p* = 0.0324^c^; *p* = 0.0206^d^
	Florfenicol	107/108 (99.1%)^c,e^	54/71 (76.1%)^d,e^	30/82 (36.6%)^c,d^	*p* < 0.0001^c,d,e^
Macrolide	Erythromycin	107/108 (99.1%)^c^	69/71 (97.2%)^d^	36/82 (43.9%)^c,d^	*p* < 0.0001^c,d^
	Tilmicosin	81/108 (75%)^c^	55/71 (75.3%)^d^	36/82 (43.9%)^c,d^	*p* < 0.0001^c,d^
Sulfonamides	Sulfadimethoxine	16/108 (14.8%)^c,e^	35/71 (49.3%)^d,e^	0/82 (0%)^c,d^	*p* < 0.0001^c,d,e^
	Trimethoprim + Sulfamethoxazole	0/108 (0%)	0/71 (0%)	0/82 (0%)	NS
Fluoroquinolones	Ciprofloxacin	108/108 (100%)^c^	69/71 (97.2%)^d^	19/82 (23.2%)^c,d^	*p* < 0.0001^c,d^
	Enrofloxacin	108/108 (100%)^c^	69/71 (97.2%)^d^	25/82 (30.5%)^c,d^	*p* < 0.0001^c,d^
	Levofloxacin	108/108 (100%)^c^	69/71 (97.2%)^d^	15/82 (18.3%)^c,d^	*p* < 0.0001^c,d^
	Moxifloxacin	107/108 (99.1%)^c^	69/71 (97.2%)^d^	18/82 (22%)^c,d^	*p* < 0.0001^c,d^
Nitrofuran	Nitrofurantoin	0/108 (0%)	0/71 (0%)	0/82 (0%)	NS
Lincosamide	Clindamycin^f^	101/108 (93.5%)^c,e^	55/71 (77.5%)^d,e^	39/82 (47.6%)^c,d^	*p* < 0.0001^c^
					*p* = 0.0025^d^
					*p* = 0.0002^e^
Pleuromutilin	Tiamulin	0/108 (0%)^c^	1/71 (1.4%)	7/82 (8.5%)^c^	*p* = 0.0024^c^
Glycopeptide	Vancomycin	0/108 (0%)	0/71 (0%)	0/82 (0%)	NS
Oxazolidinone	Linezolid	1/108 (0.9%)	0/71 (0%)	0/82 (0%)	NS

^a^Number resistant out of total isolates tested (percent resistant). ^b^Statistical significance designated at *p* < 0.05; NS, not significant. ^c,d,e^Statistical significance designated at *p* < 0.05. ^f^Inducible clindamycin.

The AMR indices for human clinical MRSA CC5-SDR isolates ranged from 0.35–0.58, which equated to phenotypic resistance to 11 and 18 antibiotics, respectively. The median AMR index observed for clinical MRSA CC5-SDR isolates was 0.48 (Figure 1). The AMR indices exhibited by clinical MRSA CC5-SDR isolates were similar to the AMR indices for human clinical MRSA CC5-NSC (ranging from 0.21–0.59; median = 0.52). Both the human clinical MRSA CC5-SDR (median = 0.48) and human clinical MRSA CC5-NSC isolates (median = 0.52) had significantly higher AMR indices than LA-MRSA CC5 isolates (median = 0.38) (*p* < 0.0001), which equated to phenotypic resistance to an average of 15 and 14.6 antibiotics respectively, compared to 11.7 antibiotics. These data demonstrate that AMR was generally less prevalent among swine associated LA-MRSA CC5 isolates than the AMR prevalence among both human clinical MRSA CC5-NSC and clinical MRSA CC5-SDR isolates.

**Figure 1:**
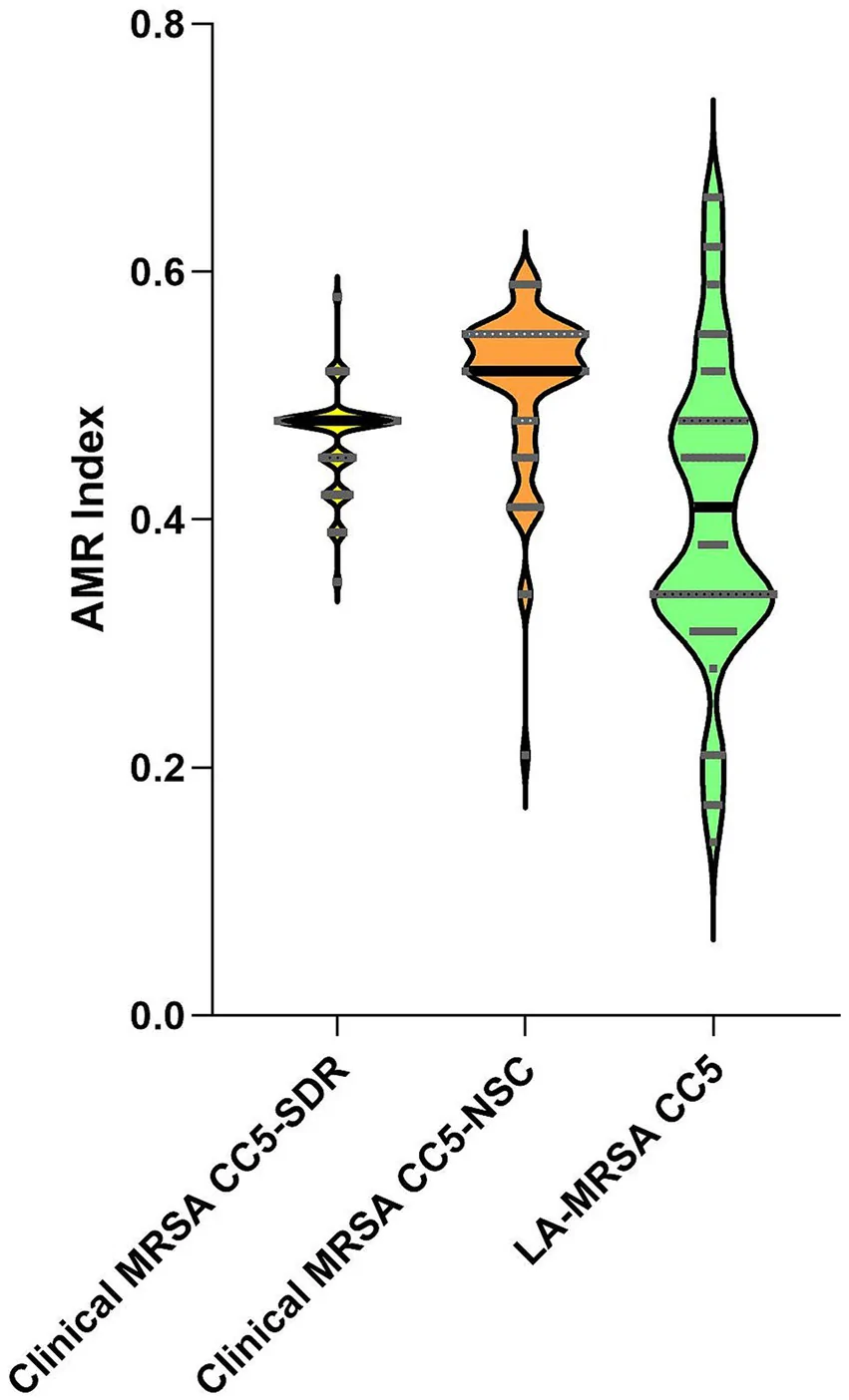
AMR index of isolates from human clinical MRSA CC5-SDR, human clinical MRSA CC5-NSC, and swine-associated LA-MRSA CC5. AMR index is defined as the proportion of the tested antibiotics to which an isolate was phenotypically resistant. The AMR index is plotted along the y-axis and each subgroup (human clinical MRSA CC5-SDR, human clinical MRSA CC5-NSC, and swine-associated LA-MRSA CC5) is shown along the *X*-axis. The human clinical MRSA CC5-SDR isolates shown in yellow (median = 0.48; bold line) and human clinical MRSA CC5-NSC isolates shown in orange (median = 0.52; bold line) had significantly higher AMR indices than LA-MRSA CC5 isolates shown in green (median = 0.38; bold line) (*p* < 0.0001).

Human clinical MRSA CC5-SDR isolates were screened for resistance to vancomycin and linezolid, which are antibiotics typically used for treating MRSA infections in hospital settings. One isolate exhibited phenotypic resistance to linezolid, and no isolates exhibited phenotypic resistance to vancomycin. Genetic determinants conferring vancomycin resistance and the multidrug resistance gene *cfr* were not found in any genomes of the clinical MRSA CC5-SDR isolates.

### Aminoglycoside resistance

Phenotypic neomycin and streptomycin resistance was not significantly different among the subsets of isolates (Table 3). In contrast, significantly lower phenotypic gentamicin resistance was observed among human clinical MRSA CC5-SDR isolates compared to both MRSA CC5-NSC isolates and LA-MRSA CC5 isolates (Table 3). Isolates were screened for genetic determinants that confer aminoglycoside resistance. Consistent with observed phenotypic resistance, the *aac(6′)-Ie/aph(2″)-Ia* gene conferring gentamicin resistance was observed significantly less in clinical MRSA CC5-SDR isolates (1.9%) compared to both MRSA CC5-SDR (12.7%) and LA-MRSA CC5 isolates (17.1%) (Table 4). The aminoglycoside resistance genes *aadD1, aph(3′)-IIIa*, *ant(6)-Ia*, and *ant(9)-Ia* were identified in statistically significant higher percentages of human clinical MRSA CC5-SDR and MRSA CC5-NSC isolates compared to LA-MRSA CC5 isolates (Table 4). Sequence analysis determined that the aminoglycoside resistance gene *aadD1* was located on different multi-resistant plasmids for the majority of human clinical MRSA CC5-SDR and MRSA CC5-NSC isolates compared to LA-MRSA CC5 isolates. No streptomycin resistance was observed among the human clinical MRSA CC5-SDR isolates and no genetic determinants conferring streptomycin resistance were found in clinical MRSA CC5-SDR isolates. One LA-MRSA CC5 isolate exhibited phenotypic resistance to streptomycin and harbored the *str* resistance gene (Table 4). One clinical MRSA CC5-SDR isolate exhibited phenotypic resistance to streptomycin, however, a *str* resistance gene was not found when the draft genome was screened for genetic AMR elements (Table 4).

**Table 4:** AMR genes identified among isolate groups.

Resistance genes	Clinical MRSA CC5-SDR^a^	Clinical MRSA CC5-NSC^a^	LA-MRSA CC5^a^	Statistics^b^
Aminoglycoside resistance				
*aac(6′)-Ie/aph(2″)-Ia*	2/108 (1.9%)^c,e^	9/71 (12.7%)^e^	14/82 (17.1%)^c^	*p* = 0.0003^c^_;_ *p* = 0.0075^e^
*aadD1*	90/108 (83.3%)^c^	62/71 (87.3%)^d^	57/82 (69.5%)^c,d^	*p* = 0.0324^c^_;_ *p* = 0.0206^d^
*aph(3′)-IIIa*	11/108 (10.2%)^c^	15/71 (21.1%)^d^	0/82 (0%)^c,d^	*p* = 0.0027^c^_;_ *p* < 0.0001^d^
*ant(6)-Ia*	15/108 (13.9%)^c^	15/71 (21.1%)^d^	1/82 (1.2%)^c,d^	*p* = 0.0027^c^_;_ *p* < 0.0001^d^
*ant(9)-Ia*	93/108 (86.1%)^c^	63/71 (88.7%)^d^	0/82 (0%)^c,d^	*p* < 0.0001^c,d^
*str*	0/108 (0%)	0/71(0%)	1/82 (1.2%)	NS
β-lactam resistance				
*blaZ*	96/108 (89.0%)^c^	58/71 (81.7%)^d^	5/82 (6.1%)^c,d^	*p* < 0.0001^c,d^
*blaPC1*	0/108 (0%)^c^	0/71 (0%)^d^	58/82 (70.7%)^c,d^	*p* < 0.0001^c,d^
Fluoroquinolone resistance				
*gyrA_*S84A	0/108 (0%)	0/71 (0%)	1/82 (1.2%)	NS
*gyrA_*S84L	108/108 (100%)^c,e^	48/71 (67.6%)^d,e^	16/82 (19.5%)^c,d^	*p* < 0.0001^c,d,e^
*gyrA_*E88A	3/108 (2.8%)	0/71 (0%)	0/82 (0%)	NS
*gyrA_*E88K	7/108 (6.5%)^c^	4/71 (5.6%)^d^	0/82 (0%)^c,d^	*p* = 0.02^c^_;_ *p* = 0.0443^d^
*gyrA_*S85P	2/108 (1.9%)	5/71 (7.0%)^d^	0/82 (0%)^d^	*p* = 0.0199^d^
*gyrA_*E88G	0/108 (0%)	1/71 (1.4%)	0/82 (0%)	NS
*parC_*E84G	43/108 (39.8%)^c^	32/71 (45.1%)^d^	0/82 (0%)^c,d^	*p* < 0.0001^c,d^
*parC_*E84K	20/108 (18.5%)^c^	10/71 (14.1%)^d^	0/82 (0%)^c,d^	*p* < 0.0001^c^; *p* = 0.0003^d^
*parC_*S80F	63/108 (58.3%)	34/71 (47.9%)	49/82 (59.8%)	NS
*parC_*S80Y	43/108 (39.8%)^c^	34/71 (47.9%)^d^	0/82 (0%)^c,d^	*p* < 0.0001^c,d^
*parE_*D432N	9/108 (8.3%)^c^	3/71 (4.2%)	0/82 (0%)	*p* = 0.011^c^
*parE_*P585S	2/108 (1.8%)	3/71 (4.2%)	0/82(0%)	NS
Macrolide, lincosamide, streptogramin resistance				
*erm*(A)	98/108 (90.7%)^c^	65/71 (91.5%)^d^	0/82 (0%)^c,d^	*p* < 0.0001^c,d^
*erm*(C)	0/108 (0%)^c^	0/71 (0%)^d^	32/82 (39.0%)^c,d^	*p* < 0.0001^c,d^
*mph*(C)	14/108 (13.0%)^c,e^	20/71 (28.2%)^d,e^	0/82 (0%)^c,d^	*p* = 0.0003^c^
				*p* ≤ 0.0001^d^
				*p* = 0.0185^e^
*msr*(A)	14/108 (13.0%)^c,e^	20/71 (28.2%)^d,e^	0/82 (0%)^c,d^	*p* = 0.0003^c^
				*p* ≤ 0.0001^d^
				*p* = 0.0185^e^
*lnu*(A)	0/108 (0%)	2/71 (2.8%)	0/82 (0%)	NS
*lnu*(B)	0/108 (0%)	0/71 (0%)	1/82 (1.2%)	NS
*lsa*(E)	0/108 (0%)	0/71 (0%)	1/82 (1.2%)	NS
*vga*(A)	0/108 (0%)^c^	0/71 (0%)^d^	80/82 (98.0%)^c,d^	*p* < 0.0001^c,d^
*vga*(E)	0/108 (0%)	0/71 (0%)	2/82 (2.4%)	NS
Tetracycline resistance				
*tet*(38)	108/108 (100%)	70/71 (98.6%)	82/82 (100%)	NS
*tet*(K)	1/108 (0.9%)	0/71 (0%)	0/82 (0%)	NS
*tet*(L)	0/108 (0%)^c^	0/71 (0%)^d^	52/82 (63.4%)^c,d^	*p* < 0.0001^c,d^
*tet*(M)	1/108 (0%)	0/71 (0%)	1/82 (1.2%)	NS
*tet*(T)	0/108 (0.9%)^c^	0/71 (0%)^d^	43/82 (52.4)^c,d^	*p* < 0.0001^c,d^

^a^Number resistant out of total isolates tested (percent resistant). ^b^Statistical significance designated at *p* < 0.05; NS, not significant. ^c,d,e^Statistical significance designated at *p* < 0.05.

### β-lactam resistance

As expected, all isolates exhibited phenotypic resistance to β- antibiotics lactam antibiotics, while genomic screening for AMR genes revealed differences in resistance genes harbored between the isolate subsets. When isolates were screened for genetic determinants that confer β-lactam resistance, the *blaPC1* gene sequence was identified in 70.7% LA-MRSA CC5 isolates. The *blaPC1* gene sequence harbored by these isolates was 100% identical to the reference *blaPC1* gene sequence allele in the NCBI AMR Gene Database (Feldgarden et al., 2019). In contrast, the *blaZ* gene harbored by the human clinical MRSA CC5-SDR (89.0%) and the MRSA CC5-NSC isolates (81.7%) was not identical to the *blaPC1* reference allele and therefore categorized as a *blaZ* family gene (Feldgarden et al., 2019).

### Fluoroquinolone resistance

Phenotypic fluoroquinolone resistance was significantly higher in both human clinical MRSA CC5-SDR and MRSA CC5-NSC isolates compared to LA-MRSA CC5 isolates for all fluoroquinolone antibiotics tested (Table 3). When isolates were screened for AMR elements, fluoroquinolone resistance was observed to be associated with mutations in *gyrA*, *parC*, or *parE* in both human clinical MRSA CC5-SDR and MRSA CC5-NSC isolates, while resistance in LA-MRSA CC5 isolates was primarily associated with mutations in both *gyrA* and *parC* (Table 3). Overall, the *gyrA* mutation gyrA_S84L was the most prevalent mutation associated with fluoroquinolone resistance. The *gyrA_S84L* mutation was (Table 4). Despite a low prevalence, the *gyrA* mutations *gyrA_*E88K and *gyrA_*S85P were identified in only human clinical MRSA CC5-SDR and MRSA CC5-NSC isolates (Table 4). Mutations in *gyrA* that were infrequently identified include gyrA_S84A, which was found in only one LA-MRSA CC5 isolate, *gyrA_*E88G, found in only one MRSA CC5-NSC isolate, and gyrA_E88A, found in only three MRSA CC5-SDR isolates (Table 4). The *parC* mutations *parC_*E84G, *parC_*E84K, and *parC_*S80Y were identified in a statistically significant higher percentage of human clinical MRSA CC5-SDR and MRSA CC5-NSC isolates compared to LA-MRSA CC5 isolates. In contrast. *parC_*S80F was found in similar percentages among all three isolate groups. Mutations in *parE* mutations were only found in human clinical MRSA CC5-SDR and MRSA CC5-NSC isolates (Table 4).

### Macrolide, lincosamide, and streptogramin resistance

Phenotypic resistance to macrolide and lincosamide antibiotics was significantly higher in human clinical MRSA CC5-SDR and MRSA CC5-NSC isolates compared to LA-MRSA CC5 isolates (Table 3). Additionally, phenotypic resistance to clindamycin was significantly higher in human clinical MRSA CC5-SDR compared to MRSA CC5-NSC isolates (Table 3). Genomic screening for AMR genes conferring resistance to MLS antibiotics revealed differences in resistance genes harbored among isolate subsets. Specifically, the MLS resistance genes *erm*(A), *mph*(C), and *msr*(A) were only found in human clinical MRSA CC5-SDR and MRSA CC5-NSC isolates. Additionally, the macrolide antibiotic inactivation gene *mph(C)*, and *msr(A)*, encoding a streptogramin and macrolide efflux pump were both identified in a statistically higher percentage of human clinical MRSA CC5-NSC isolates compared to both MRSA CC5-SDR and LA-MRSA CC5 isolates (Table 4). There was no correlation identified between the significantly higher phenotypic resistance to clindamycin in human clinical MRSA CC5-SDR isolates compared to MRSA CC5-NSC isolates and the MLS resistance genes harbored by these isolates. Focusing on LA-MRSA CC5 isolates, the MLS resistance gene *erm*(C) and the *vga*(A) gene, which functions as an efflux pump with activity toward streptogramin A antibiotics and lincosamide antibiotics were only found in LA-MRSA CC5 isolates (Table 4). Sequence analysis determined that the *erm(C)* gene was located on a small 2.4 kb plasmid harbored by the LA-MRSA CC5 isolates (32/82, 39%). The *erm(C)* gene was the only AMR gene contained on this small plasmid (Supplementary Figure 1). Additionally, the plasmid was not identified in any of the human clinical MRSA CC5-SDR and MRSA CC5-NSC isolates.

### Tetracycline resistance

Phenotypic resistance to tetracycline was predominantly observed in LA-MRSA CC5 isolates (Table 3). Specifically, 79.3% of LA-MRSA CC5 isolates were phenotypically resistant to all tetracycline antibiotics tested. In contrast, only two human MRSA CC5-SDR isolates were phenotypically resistant to the tetracycline antibiotics tested and none of the human MRSA CC5-NSC isolates exhibited phenotypic tetracycline resistance (Table 3). Screening isolates for AMR elements conferring tetracycline resistance revealed that all human MRSA CC5-SDR isolates and 98.6% of human MRSA ST5-NSC isolates harbored a *tet (38)* chromosomal gene despite not exhibiting phenotypic resistance except for two isolates. The high prevalence of the *tet (38)* gene did not correlate with the lack of phenotypic tetracycline resistance exhibited by these isolate subsets. The *tet (38)* gene was also identified in all LA-MRSA CC5 isolates. In addition to the *tet (38)* gene, tetracycline resistance genes *tet(L)* and *tet(T)* were identified in LA-MRSA CC5 isolates. The *tet(L)* and *tet(T)* genes harbored by the LA-MRSA CC5 isolates were located on a plasmid that also contained the aminoglycoside resistance gene *aadD*.

### Genotypic resistance profile

All of the AMR genetic elements harbored by each isolate were used to generate a hierarchical clustered distance matrix heatmap representing isolate similarity based on the presence or absence of AMR genetic elements (Figure 2). Two clades were observed, one containing both of the human clinical MRSA CC5-SDR and MRSA CC5-NSC isolates, and second clade comprised of the swine associated LA-MRSA CC5 isolates (Figure 2). One human clinical MRSA CC5-SDR isolate, UCSF14436, was observed grouped within the LA-MRSA CC5 isolate clade. This isolate had a low AMR Index of 0.21 and found to harbor only four antimicrobial resistance genes (*tet (38), mecA, blaZ, and fosB*), which likely contributed to the placement of this isolate within the swine associated clade.

**Figure 2:**
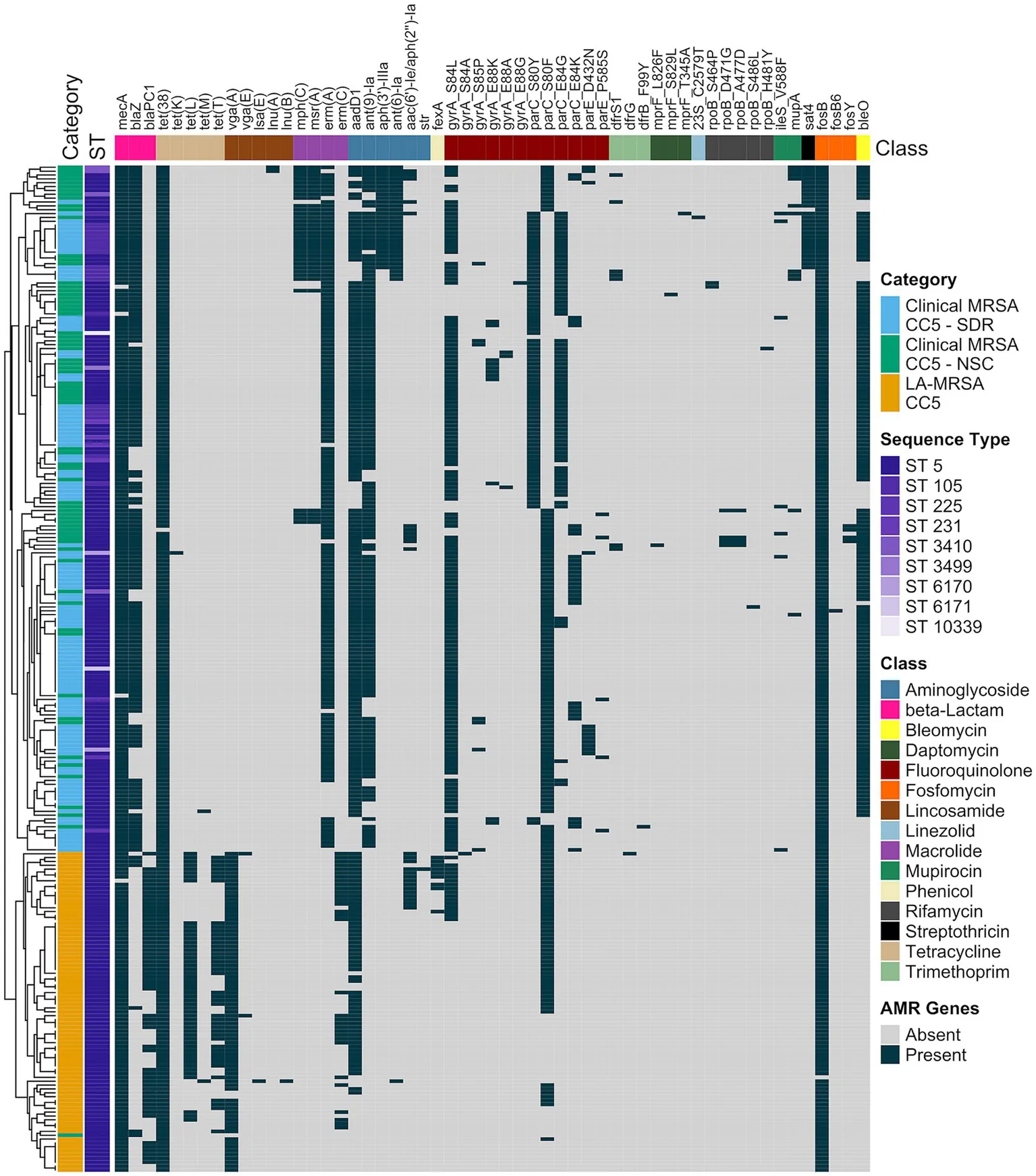
Hierarchical cluster heatmap displaying the relatedness of MRSA CC5 isolates based on the presence or absence of AMR elements. Isolates are grouped based on hierarchical clustering using a complete linkage method with Euclidean distance. AMR genetic elements are listed at the top. Isolate subgroup or category and ST are provided at the left side of heatmap and represented using the colors shown left of heatmap. AMR genetic elements are listed as AMR genes designated as present or absent and are represented using the colors shown right of heatmap. Antibiotic class for each AMR genetic element is shown at the top of heatmap and represented using the colors shown at left of heatmap.

Overall, the differences in the AMR resistance genes from each of the above-mentioned antibiotic classes are easily observable and distinct between the clade separation of the human clinical and swine associated isolate subgroups. Other distinct differences in the AMR genes harbored by the subsets of isolates include the bleomycin resistance gene *bleO* and streptothricin resistance gene *sat4*, which were only found in clinical MRSA CC5-SDR and MRSA CC5-NSC isolates. While the mupirocin resistance genes *mupA and ileS_V588F* were found in a lower number of isolates, they were only found in human clinical MRSA CC5-SDR and MRSA CC5-NSC isolates. Additionally, the phenicol resistance gene *fexA* was only found in LA-MRSA CC5 isolates.

### Multi-resistant plasmids

Evaluation of the draft genome assemblies for the clinical MRSA CC5-SDR isolates revealed several plasmids that contained two or more AMR genes. The smallest of the identified plasmids was initially identified in isolate ASR1-64. This plasmid was approximately 5 kb in size and contained the aminoglycoside resistance gene *aaD1* along with the bleomycin resistance gene *bleO* (Supplementary Figure 2). This plasmid was similar to the well characterized *S. aureus* plasmid pUB110 (Gryczan et al., 1978), with the major difference being the insertion of an IS6-like element IS257 family transposase adjacent to the *aadD1* gene. This plasmid, with minor sequence variations, was identified in the majority of the human clinical MRSA CC5-SDR isolates (89/108, 82.4%). Two additional isolates were identified in which the plasmid sequence was present in a much longer contig, indicating that like pUB110, this plasmid may be able to integrate into the chromosome (Dubin et al., 1991). This plasmid, with minor sequence variations, was additionally identified in the majority of the human clinical MRSA CC5-NSC isolates (64/71, 90.1%). However, the plasmid was not identified in any of the LA-MRSA CC5 isolates.

Another plasmid approximately 21.5 kb in size was identified in four of the human clinical MRSA CC5-SDR isolates (ASR1-71, ASR1-55, ASR1-58, and ASR2-31) that contained numerous resistance genes, including the β-lactamase resistance genes *blaZ*, *blaI, and blaRI,* macrolide resistance genes *msr(A)* and *mph(C)*, the streptothricin resistance gene *sat4*, a cadmium resistance transporter gene *cadD*, and aminoglycoside resistance genes *aph(3′)-IIIa*, and *ant(6)-Ia* (Supplementary Figure 3). The *ant(6)-Ia* gene was annotated as a pseudogene due to an internal stop codon. The draft genome sequences of two other isolates (ASR1-29 and ASR2-16) were additionally found to contain a contig with the majority of this 21.5 kb plasmid sequence. However, the contig sequence for these isolates did not circularize, which prevented the definitive categorization of these contig sequences as the same plasmid. Evidence for the presence of this plasmid was found in human clinical MRSA CC5-SDR isolates (14/108, 13%) and human clinical MRSA CC5-NSC (14/71, 19.7%). No evidence for the presence of this plasmid was found in any of the LA-MRSA CC5 isolates.

A third plasmid identified in 49 human clinical MRSA CC5-SDR isolates (49/108; 45.3%) and (21/71; 29.6%) of human clinical MRSA CC5-NSC was approximately 27 kb in size and contained the β-lactamase resistance genes *blaZ*, *blaI*, *blaRI.* In addition to the β-lactamase resistance genes, the plasmid additionally harbored the enterotoxin genes *ser*, *sej*, and *sed* (Supplementary Figure 4). Upon further examination, the *ser* gene sequence was annotated as a pseudogene due to a frameshift in the plasmid sequence for 4 of the human clinical MRSA CC5-SDR isolates and the *sed* gene sequence was annotated as a pseudogene due to a frameshift in the plasmid sequence for 7 of the human clinical MRSA CC5-SDR isolates. None of the plasmid sequences contained both *ser* and *sed* gene sequences annotated as a pseudogenes. The draft genome sequences of an additional 27 isolates were identified as having a contig that contained the majority of this 27 kb plasmid sequence. Similar to the previous plasmid description, the contig sequence for these isolates did not circularize, which prevented the categorization of these contig sequences as the same plasmid. This plasmid was not found in any LA-MRSA CC5 isolates.

A fourth multi-resistance plasmid, approximately 49 kb in size, was identified in human clinical MRSA CC5-SDR isolate ASR1-3. The plasmid sequence contained the β-lactamase resistance genes *blaZ*, *blaI*, and *blaRI*, the quaternary ammonium resistance genes *qac* and *qacR*, and the mupirocin resistance gene *mupA*, which was annotated as pseudogenes due to a frameshift (Supplementary Figure 5). The plasmid additionally harbored the enterotoxin genes *ser*, *sej*, and *sed*.

A fifth plasmid, approximately 24.7 kb in size, was identified in the human clinical MRSA CC5-SDR isolate ASR2-8. The plasmid sequence contained the β-lactamase resistance genes *blaZ, blaI,* and *blaRI*, the cadmium resistance gene *cadD*, and the arsenic resistance operon *arsRBC* (Supplementary Figure 6). The nucleotide sequence of the *arsRBC* operon was found to be 99.7% identical to the *arsRBC* operon from *S. aureus* multi-resistance plasmid pI258 (Ji and Silver, 1992). Additionally, this plasmid also contained a putative lactococcin 972 family bacteriocin gene cluster.

The majority of LA-MRSA CC5 isolates (56/82, 68.3%) harbored a plasmid, approximately 9.3 kb in size, which contained the aminoglycoside resistance gene *aadD1*, and the tetracycline resistance genes *tet(L)* and *tet(T)* (Supplementary Figure 7). This plasmid was not identified in any of the human clinical MRSA CC5-SDR and MRSA CC5-NSC isolates.

## Discussion

The emergence of MRSA in global livestock settings has become a focal point in discussions regarding the role of antibiotic use in food-animal production. The appearance of ST5 isolates within United States swine herds has intensified concerns regarding the capacity of LA-MRSA to serve as a reservoir for AMR dissemination beyond the agricultural sector. In this report human clinical MRSA CC5 isolates obtained from swine-dense regions were evaluated for *czrC* gene prevalence and the prevalence and diversity of AMR phenotypes and AMR genetic elements. These results were then compared to the *czrC* gene prevalence, phenotypic resistances, and AMR genetic elements harbored among human clinical MRSA CC5 isolates obtained from humans with no swine contact and swine-associated LA-MRSA CC5 isolates. Comparisons across all three isolate subgroups provided a comprehensive evaluation of the potential for LA-MRSA CC5 isolates to act as a likely source of MGEs harboring AMR determinants.

When *czrC* gene prevalence was evaluated, only one clinical MRSA CC5-SDR isolate was found harboring the *czrC* gene. The extremely low *czrC* gene prevalence observed among the clinical MRSA CC5-SDR isolates was similar to the low *czrC* gene prevalence observed among LA-MRSA CC5 isolates. Whereas over one-fifth of the clinical MRSA CC5-NSC isolates contained the *czrC* gene. Collectively, the data reported here indicate that any potential co-selection associated with in feed zinc supplementation has not contributed to the persistence or prevalence of MRSA CC5 isolates in swine dense agricultural areas and are in agreement with previous findings (Hau et al., 2017). The low prevalence of the *czrC* gene identified among CC5 isolates is in agreement with earlier studies suggesting that the distribution of *czrC* gene-associated zinc resistance in MRSA is strongly influenced by lineage (Hau et al., 2017).

Overall, phenotypic AMR was significantly lower among swine associated LA-MRSA CC5 isolates compared to the AMR prevalence among both human clinical MRSA CC5-NSC and clinical MRSA CC5-SDR isolates. Likewise, the swine associated LA-MRSA CC5 isolates harbored significantly fewer AMR genetic elements compared to the AMR genetic elements harbored by both human clinical MRSA CC5-NSC and clinical MRSA CC5-SDR isolates. The divergence in AMR genetic elements between human-associated and swine-associated isolates contributed to the clustering of the isolate subgroups into two distinct phylogenetic clades. One clade comprised both human clinical MRSA CC5-SDR and MRSA CC5-NSC isolates, and the second clade comprised the swine associated LA-MRSA CC5 isolates. Additionally, differences among the isolate subgroups were observed not only in the AMR genetic elements they carried but also in the genomic locations of these elements, such as their presence on plasmids. Specifically, different plasmids containing different constellations of AMR genes were identified among isolate subgroups. For instance, plasmids containing enterotoxins were only observed among both human clinical MRSA CC5-SDR and MRSA CC5-NSC isolates but not in swine associated LA-MRSA CC5 isolates. These findings provide additional evidence that animal- and human-associated CC5 MRSA reservoirs are phylogenetically distinct (Hau et al., 2018a,b).

Differences in the AMR genetic elements that confer specific resistances were additionally identified among the isolate subgroups. For example, while all isolates exhibited β-lactamase, the β-lactamase resistance gene *blaPC1* gene was only identified among the LA-MRSA CC5 isolates. Focusing on AMR elements conferring fluoroquinolone resistance, mutations in *parE and gyrA_*E88K and *gyrA_*S85P mutations were only identified in the human-associated isolate subgroups. The AMR elements *erm(A)*, *mph(C)*, and *msr(A)*, conferring MLS resistance were also only found in human-associated subgroups. In contrast, the MLS resistance genes *vga(A)* and *erm(C)* were found only among isolates belonging to the swine-associated subgroup. Additionally, the majority of LA-MRSA CC5 isolates harbored the *erm(C)* gene on a small plasmid, with *erm(C)* representing the only antimicrobial resistance gene encoded by the plasmid.

While phenotypic tetracycline resistance was predominately exhibited by the swine-associated subgroup LA-MRSA CC5 isolates, tetracycline resistance gene *tet (38)* chromosomal gene was highly prevalent among isolates in every group. The high prevalence of the *tet (38)* gene among human clinical MRSA CC5-SDR and MRSA CC5-NSC isolates did not correlate with the lack of phenotypic tetracycline resistance exhibited by these isolate subsets. This may be due to the expression of *tet (38)* being strongly regulated by repressor proteins, such as MgrA, which typically require specific environmental stresses or regulatory mutations to achieve significant resistance (Truong-Bolduc et al., 2015). Although *tet (38)* is widely prevalent among many types of *S. aureus* strains, high-level tetracycline resistance is more commonly associated with the acquisition of mobile genetic elements, such as plasmids (Brdova et al., 2024; Vestergaard et al., 2019). Consistent with the phenotypic data, tetracycline resistance in LA-MRSA CC5 isolates was associated with the presence of *tet(L)* and *tet(T)*, both of which were carried on a plasmid that also encoded the aminoglycoside resistance determinant *aadD*.

To continue addressing public health concerns regarding the potential dissemination of AMR by CC5 LA-MRSA isolates beyond agricultural settings, human clinical MRSA CC5 isolates obtained from swine-dense regions were evaluated for *czrC* gene prevalence and AMR prevalence and diversity. These results were then compared to the *czrC* gene prevalence and AMR prevalence and diversity harbored among human clinical MRSA CC5 isolates obtained from humans with no swine contact and swine-associated LA-MRSA CC5 isolates. Across all three isolate subgroups, the *czrC* gene was detected at a low frequency, further supporting the concept that zinc resistance in MRSA is primarily shaped by lineage-specific factors. Swine-associated LA-MRSA CC5 isolates exhibited resistance to fewer antibiotics than human-associated isolate subgroups. Additionally, separate and distinct AMR genetic determinants, including plasmid encoded AMR elements, were harbored by human-associated and swine associated CC5 isolate groups. Collectively, the data in this report such that, among the CC5 isolates examined, human and swine-associated CC5 MRSA reservoirs are distinct, and the swine reservoir is not a likely source for AMR dissemination beyond the agricultural sector.

## Data Availability

The datasets presented in this study can be found in online repositories. The names of the repository/repositories and accession number(s) can be found in the article/Supplementary material.

## Footnotes

1
https://www.bioinformatics.babraham.ac.uk/projects/fastqc/

2
https://github.com/lh3/seqtk

3
http://mira-assembler.sourceforge.net/docs/DefinitiveGuideToMIRA.html

4
https://pubmlst.org

## Supplementary material

SUPPLEMENTARY FIGURE 1 LA-MRSA CC5 macrolide resistance plasmid. A small plasmid harboring the *erm(C)* macrolide resistance gene was found in 32/82 (39%) of LA-MRSA CC5 isolates. A representative sequence from ISU886 is shown.

SUPPLEMENTARY FIGURE 2 Small plasmid containing *aadD1* and *bleO*. The small plasmid containing the aminoglycoside resistance gene *aadD1* and bleomycin resistance gene *bleO* was found in 89/108 (82.4%) of human clinical MRSA CC5-SDR isolates and 64/71 (90.1%) of human clinical MRSA CC5-NSC isolates. Representative examples of this plasmid can be found in isolates ASR1-64 and UCI11.

SUPPLEMENTARY FIGURE 3 Multi-resistance plasmid. Evidence for the presence of this plasmid encoding resistance genes to multiple antibiotic classes was found human clinical MRSA CC5-SDR isolates (14/108, 13%) and human clinical MRSA CC5-NSC (14/71, 19.7%). No evidence for the presence of this plasmid was found in any of the LA-MRSA CC5 isolates. This plasmid carries genes for β-lactamase resistance (*blaZ*, *blaR1*, *blaI*), macrolide resistance (*msr(A)*, *mph(C)),* aminoglycoside resistance (*aph(3′)-IIIa*, *ant(6)-Ia*), and streptothricin resistance (*sat4*). It also harbors the *cadD* cadmium resistance transporter. A representative example of this plasmid can be found in ASR1-71.

SUPPLEMENTARY FIGURE 4 β-lactamase and enterotoxin-containing plasmid. A plasmid carrying the β-lactamase resistance genes *blaZ*, *blaR1*, and *blaI*, the enterotoxin genes *sed*, *sej*, and *ser*, and the cadmium resistance transporter gene *cadD* was identified in human clinical MRSA CC5-SDR isolates (49/108; 45.3%) and (21/71; 29.6%) of human clinical MRSA CC5-NSC. This plasmid was not found in any LA-MRSA CC5 isolates. In a small number of isolates, 1 or more of the enterotoxin genes were annotated as frameshifted pseudogenes. A representative sequence from ASR1-18 is shown.

SUPPLEMENTARY FIGURE 5 β-lactamase and enterotoxin-containing plasmid. A β-lactamase and enterotoxin-containing plasmid was found in human clinical MRSA CC5-SDR isolate ASR1-3. This plasmid carries the resistance to quaternary ammonium genes *qac* and *qacR*. A frameshifted mupirocin resistance gene (*mupA*) is also present on this plasmid.

SUPPLEMENTARY FIGURE 6 β-lactamase and arsenic resistance-containing plasmid. Isolate ASR2-8 was found to carry a plasmid containing the β-lactamase resistance genes (*blaZ*, *blaR1*, and *blaI*), the cadmium resistance transporter *cadD*, the arsenic resistance operon *arsRBC*, and a putative bacteriocin operon (containing genes for the bacteriocin precursor protein and the transmembrane immunity protein).

SUPPLEMENTARY FIGURE 7 LA-MRSA CC5 tetracycline resistance plasmid. A plasmid encoding the tetracycline resistance genes *tet(L)* and *tet(T)* along with the aminoglycoside resistance gene *aadD1* was identified in LA-MRSA CC isolates (56/82, 68.3%). A representative example from ISU952 is illustrated.
